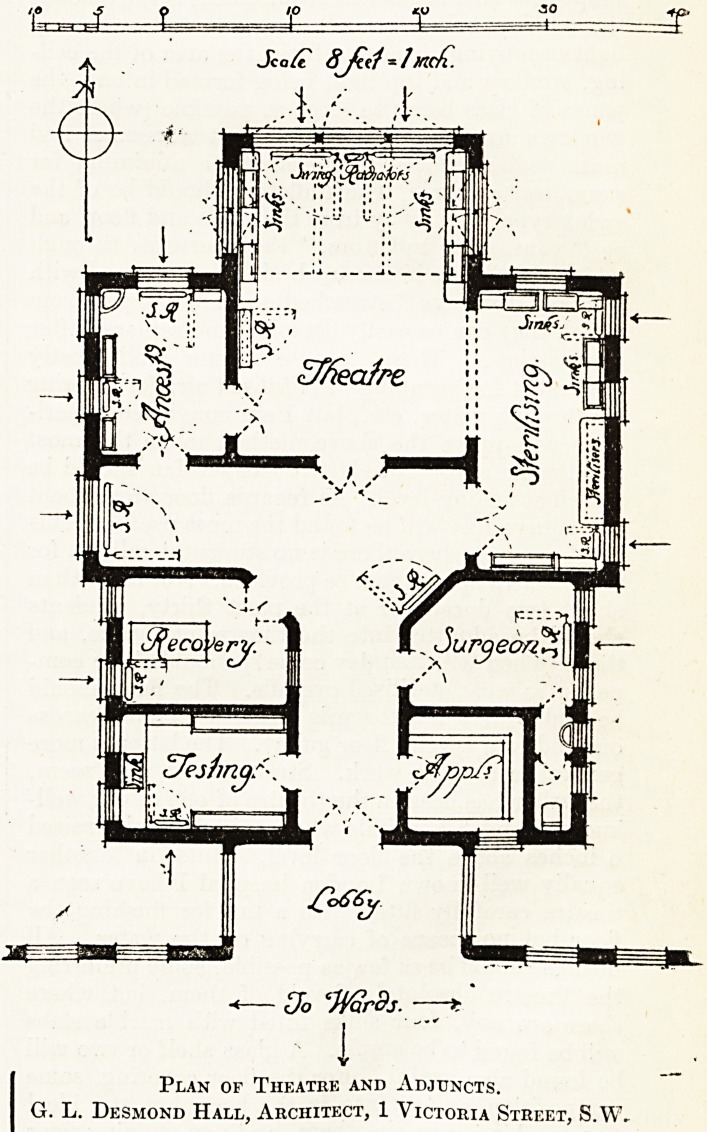# The Planning of the Modern Operating Theatre Unit

**Published:** 1912-07-13

**Authors:** G. L. Desmond Hall

**Affiliations:** Architect.


### HOSPITAL ARCHITECTURE AND CONSTRUCTION.

[Communications on this subject should be marked "Architecture" in the left-hand top corner of the envelope.]

### The Planning of the Modern Operating Theatre Unit.

By G. L. DESMOND HALL, Architect.

It has often been said that if a hospital is adequately to fulfil its function it must be planned by the architect in conjunction with the medical superintendent. This is especially true in the case of the operating theatre unit, only here it is not the superintendent alone who should be consulted, but the surgeons who would use the theatre, while the anæsthetist also will have his opinion in the matter. There is, in addition, another person whom it would pay the architect to consult, who knows as much about theatre work as the surgeon—who, in fact, knows more of the practical working of a theatre unit; and that person is the sister in charge of the theatre. This sister is generally an intelligent person of thorough and sound experience, and should be listened to by the architect with the greatest respect. She, as it were, sets the scenery ready for the great actor, namely, the surgeon, who only appears when everything is fully prepared, and then, having done his work, retires, leaving the theatre sister to change the scenery and reset the stage for the next act. In consulting the surgeons of a hospital about some theatre fitting one will generally find that they will spend hours in committee arguing and discussing the merits or demerits of some very minute and probably unnecessary detail, with perhaps the result that some elaborate and costly fitting will be installed which will be proved after a time to be impracticable, and will have to be removed, thereby throwing the theatre out of use pro tem., causing the other theatres (and probably the small one intended solely for minor operations) to be doubly worked, to say nothing of the cost, nowadays such an ever-increasing factor in hospital administration. Now, if the theatre sister were to be consulted, I am confident that much unnecessary delay and cost would be avoided. On the subject of cost much has been written, so I will not take up valuable room on this matter, but merely remark that modern surgery requires the strictest asepsis, and it is impossible to obtain this without the employment of the best materials in the construction of the theatre and its adjuncts, and the best materials and high cost are synonymous.

In choosing the position of the theatre unit there are several factors which should be taken into consideration. The most important of all is light. The hospital architect is told that "theatres should have a north, or as near as possible a north, light," which rule must, of course, be strictly adhered to, but the expert architect will alter the "should, etc.," into "must have a north light," deleting the rest of the sentence. In fact, the position of this unit is practically the keynote to the whole block plan. Needless to say, the theatre should not be placed so that patients in any of the pavilions can see into it from without. On very restricted sites, or on sites somewhat crowded by the inevitable future extension, it is often impossible to prevent the theatre from being overlooked, in which case obscured glass has to be used, which has its obvious disadvantages. In a certain London hospital I am acquainted with I am told that enthusiastic probationers often watch operations from the windows of their rooms, the theatre windows only being obscured for about three-quarters of their height. The ideal position for the theatre unit is immediately to the north of the surgical wards, disconnected from the closed-in corridor

Plan of Theatre and Adjuncts.
G. L. Desmond Hall, Architect, 1 Victoria Street, S.W.

or covered way by a cross-ventilated lobby, and should be on the ground floor (nothing, of course, being built over it), with a lift serving all floors in close proximity. The greatest care should be taken to protect the patient from exposure to draughts during his transit to and from the theatre, and I therefore consider that, at any rate on the surgical side, all connection between the pavilions and theatre should be by the closed-in corridor, and not by the covered way. The ideal shape of a theatre is the oval, if it were not for the fact that fittings take up such a lot of room and the cost of construction is so great. A very favourite form is an oblong with a projecting bay, but this adds to the cost of construction, at the same time giving a very large glass area in proportion to the size of the theatre, and when one takes into consideration the elevated temperature which must be maintained during an operation, it will readily be seen that the size of the radiators would have to be proportionate—another addition to the cost. Hence we arrive at the conclusion that the glass area in a theatre should be reduced to the minimum consistent with efficient lighting. The shape that best fulfils this requirement is the oblong, the window being formed along one end and a top light occupying about two-fifths the area of the ceiling, window and top light being formed in one, the panes of glass being as large as possible (where the windows are made to open), so that transomes and mullions be reduced to the absolute minimum for structural safety. The radiators should be of the swing type, well away from the walls and floor, and be "ventilating radiators," the apertures through which they draw in the fresh air being covered with gauze "stockings" stretched on light frames, from which they can be easily detached and replaced after sterilisation. There are elaborate and costly apparatus for pumping in sterilised air, for filtering air through water, etc., but I am convinced experience will prove the above method to be the most practicable one. An electric extract fan should be placed at ceiling level. As regards floor area, about 420 square feet will be found the most useful. This is, of course, where there is no students' gallery, for which extra space must be provided. Not more than about two dozen, or at the most thirty, students should be admitted into the theatre at a time, and these (when will that day come?) should all be compelled to wear sterilised overalls. The floor should be laid with a fall towards either a channel across one end or a special floor gulley. The latter is more general in modern work. Strange as it may seem, the writer has been in the theatre of one of our well-known London hospitals where this channel is raised 6 inches above the floor level, while in another equally well-known London hospital I have seen a theatre carefully fitted with a tap for flushing the floor but no means of carrying off the water. All fittings should be as few as possible, some preferring the theatre absolutely devoid of them, but where there are any, four sinks fitted with marble slabs will be found to be ample. A glass shelf or two will be found also useful. For the floor covering, some composition as terrazzo is the best, but the ideal non-cracking, non-absorbent, and non-staining floor covering has yet to be invented. It is hardly necessary to state that all corners must be rounded. The height need only be lofty enough to give an "airy" appearance. One must not forget the fact that several people will be working together in an elevated temperature for some time, so that a fairly large number of cubic feet per person must be provided. The rooms immediately adjoining and in direct communication with the theatre are the anæsthetising and sterilising rooms. There must, of course, be a separate door giving access to the theatre from the lobby, as it is obviously objectionable that the only means of entrance to the theatre should be by way of one of these rooms. The anæsthetising-room should be cut off by swing doors, while the sterilising-room should lead directly out of the theatre through a wide open archway. Some surgeons prefer that this room should also be cut off by swing doors, maintaining that the noise made by "dishing up" instruments—the sudden noise caused by one being accidentally dropped, for instance—is apt to disconcert them while operating, but the open arch is the more popular of the two. Doorways, which should be as few as possible, should be so planned that all traffic is confined to the rear of the theatre, leaving the front part (near the light) perfectly free for the table. In the sterilising-room there should be two or three ample sinks with marble slabs for washing up, some glass shelves, a glass instrument cabinet, and a "bench" for sterilisers, with an enamelled-iron hood and flue over for carrying off the heat, etc. Here floor space is of great value. The anæsthetising-room need not be very large, but in planning this room it must be remembered that space must be provided for the anæsthetist and probably his assistant, at least two nurses and two ambulances. The patient enters head foremost, in which position he remains while the anæsthetic is being administered, so that he can be taken into the theatre feet first. Thus the correct position for the window is facing the door. A sink and two cabinets for anæsthetising appliances, etc., are all the fittings required. An extract fan to clear the air of fumes is essential. This room should only communicate with the lobby and the theatre. There seems great diversity of opinion as to whether a recovery room should form part of the adjuncts to the theatre or not. It is a thing which cannot easily be added, and if, moreover, all the beds in the main ward and specials were occupied, it would be exceedingly difficult to deal with an acute case of collapse. I therefore consider the recovery-room should be provided. There should be no communication between this room and the theatre, except *viâ* the lobby. A retiring-room for the surgeon, with lavatory adjoining, and a room for appliances complete the theatre unit, but if this unit is to be absolutely up to date there should be, not of necessity in the actual unit itself but closely adjacent thereto, a small laboratory, the idea being that the surgeon can take a small "cutting" from the field of operation and send it there, where it can be examined by the biologist, and a report obtained in a few minutes, during which time the open wound is covered with sterilised materials.

In conclusion, it must be stated as a *sine qua non* that if this unit is to be worked on truly economical lines it must be complete in every respect, and above all everything must be practicable and thorough.

## Figures and Tables

**Figure f1:**